# Genetic and constitutional factors are major contributors to *substantia nigra* hyperechogenicity

**DOI:** 10.1038/s41598-017-07835-z

**Published:** 2017-08-02

**Authors:** Juan F. Vázquez-Costa, José I. Tembl, Victoria Fornés-Ferrer, Fernando Cardona, Lluis Morales-Caba, Gerardo Fortea, Jordi Pérez-Tur, Teresa Sevilla

**Affiliations:** 1 Neuromuscular Research Unit, Instituto de Investigación Sanitaria la Fe (IIS La Fe), Valencia, Spain; 2 ALS Unit, Department of Neurology, Hospital Universitario y Politécnico La Fe, Valencia, Spain; 3 Centro de Investigación Biomédica en Red de Enfermedades Raras (CIBERER), Valencia, Spain; 4 Neurosonology Laboratory, Department of Neurology, Hospital Universitario y Politécnico La Fe, Valencia, Spain; 5 Biostatistics Unit, Instituto de Investigación Sanitaria la Fe (IIS La Fe), Valencia, Spain; 6 Laboratory of Molecular Genetics, Institut de Biomedicina de València-CSIC, Valencia, Spain; 7 Centro de Investigación Biomédica en Red de Enfermedades Neurodegenerativas (CIBERNED), Valencia, Spain; 8 Unidad mixta de Neurología y Genética, Instituto de Investigación Sanitaria la Fe (IIS La Fe), Valencia, Spain; 9 Department of Medicine, University of Valencia, Valencia, Spain

## Abstract

Hyperechogenicity of *substantia nigra* (SNh) is a frequent finding in amyotrophic lateral sclerosis (ALS), Parkinson’s disease (PD) and other movement disorders (MD) patients, but its meaning is unclear. To ascertain the contribution of different factors to SNh area, we measured it in 108 ALS, 102 PD, 91 other MD patients and 91 healthy controls. Demographical data were collected in all patients and controls. In ALS patients, we also recorded clinical variables, performed genetic analysis and measured baseline levels of ferritin. After family history and genetic testing, ALS patients were classified as familial (15) or sporadic (93). ALS, PD and other MD patients had a larger SNh area than controls. Left SNh and male gender, but not age, associated with larger SNh area in both patients and controls. Familial ALS patients showed larger SNh area than sporadic ones and familial ALS was the only clinical variable in the multivariate analysis to be associated with larger SNh area in ALS patients. Our results suggest that SNh associates with genetic and constitutional factors (male gender, handedness), some of which predispose to certain neurodegenerative diseases. This evidence supports the idea of SNh as an inborn marker of unspecific neuronal vulnerability.

## Introduction

Amyotrophic lateral sclerosis (ALS) is a neurodegenerative disease clinically characterised by progressive weakness, and by signs of upper (UMN) and lower motor neuron (LMN) impairment. Three different phenotypes can be distinguished according to the degree of clinical impairment of UMN and LMN^1^: classical ALS (cALS), primary lateral sclerosis (PLS) and progressive muscular atrophy (PMA). Pathologically, ALS is characterised by TDP-43 aggregates found in degenerating UMN and LMN^1^. However, TDP-43 deposits and a variable degree of neuronal loss can be found far beyond motor neurons in many ALS patients; e.g., *substantia nigra* (SN) in about 50% of them^2, 3^. The extension of these deposits probably accounts for the presence or absence of extra-motor features, such as cognitive or behavioural impairment in ALS patients^1^.

By means of transcranial sonography (TCS), a larger area of increased echogenicity (hyperechogenicity) in SN has been found in patients with different neurodegenerative diseases (especially in Parkinson’s disease, PD) compared to controls^4^. This SN hyperechogenicity (SNh) is thought to reflect increased iron deposits^5^. Although its exact meaning remains unknown, it has been proposed to be a marker of SN degeneration or vulnerability^5^. Recently, SNh has been reported in 50–70% of cALS patients^6–9^, but whether in ALS this finding also reflects vulnerability of the nigrostriatal system or the pathologic spread of the disease to the SN is not known. Moreover, SNh has not been studied in other phenotypes (PMA or PLS) or in familial ALS.

The aim of this study was to evaluate the SNh in ALS patients (including PMA and PLS) compared to movement disorders (MD) patients and healthy controls. We also aimed to evaluate the contribution of demographical, clinical, biochemical and genetic factors to SNh in patients and controls.

## Methods

### Subjects and definitions

For this cross-sectional study, patients diagnosed with classical ALS (cALS), PMA or PLS who came to our ALS Unit between February 2014 and September 2016 and gave written informed consent, were recruited. Patients are routinely evaluated by the same neurologist (JFVC), and demographical and clinical data are prospectively recorded in a database. The cALS patients met the El Escorial revised criteria of possible, probable or definitive ALS^10^. PMA was defined as a progressive isolated impairment of LMN in at least two regions^11^, and PLS as a progressive isolated impairment of UMN in at least one region other than the lumbar region^12^.

For comparison purposes, we included a previously reported cohort of subjects without neurodegenerative diseases^13^. We also included patients with MD in whom an increased SNh area had been previously reported^14–16^: PD, atypical parkinsonism, vascular parkinsonism and essential tremor. The data of a subset of these patients have also been previously published^17^.

### Family history and genetic analysis

ALS patients were systematically asked for family history of ALS, dementia or parkinsonism of any kind in first- or second-degree relatives. When the history of dementia was compatible with frontotemporal dementia (FTD), medical records of this relative were reviewed (whenever available). Patients were categorised as familial ALS (fALS) whenever they met the criteria for possible, probable or definite ALS^18^, or as sporadic ALS (sALS) when not. Both sALS and fALS patients were screened for *C9ORF72* with repeat primed PCR, as previously reported^19^. In those fALS patients not carrying a *C9ORF72* expansion, *SOD1*, *TARDBP* and *FUS* genes were subsequently analysed by Sanger sequencing. After the genetic analysis, fALS grades were appropriately reassessed^18^.

### Clinical and analytical variables

Age, gender, time of symptom onset, region of onset (bulbar and spinal), side of onset (or side predominance in bulbar onset cases) and body mass index before disease onset (premorbid BMI) were recorded for all ALS patients.

ALS patients were examined at the time of recruitment, and the following items were recorded: parkinsonism signs, disability (ALSFRS-R)^20^, and degree of UMN impairment (UMN score) for a maximum of 16^21^.

Executive and behavioural examination were performed in a subset of 100 patients. Executive function was assessed with digit span; verbal fluency index^22^ and stroop test in patients without relevant bulbar symptoms; and trail A and B in patients without symptoms in the dominant hand. Patients were studied with at least two different executive tests, depending on their disability. Mild executive impairment was diagnosed according to current criteria^22^. Relatives were systematically asked for behavioral symptoms following Rascovsky criteria and mild behavioural impairment and FTD were diagnosed according to current criteria^22^. Moreover, 60 patients were assessed with the Spanish validated version of the Frontal Systems Behaviour Scale (FrSBe)^23^, fulfilled by the caregiver. The FrSBe has three subscales: apathy, disinhibition, and executive dysfunction. For the present study, we have considered the Z-scores of the post-illness forms.

The basal (within two months from recruitment) levels of ferritin were determined in 98 patients by immunoturbidimetry on a Beckman Coulter AU 5400 according to manufacturer’s instructions.

### TCS examination

A TCS examination was performed by an expert neurologist sonographer (JIT), who was blind to the clinical data, with the same ultrasound system (Toshiba Aplio XG, Tokyo, Japan 2008) equipped with a 2.5 MHz phased-array transducer to obtain B-mode images through a temporal acoustic bone window. Right and left SNh were obtained and measured by this examiner, as previously published^13^.

### Statistical analysis

Differences in the right and left SNh areas in patients and controls (age- and gender-adjusted) were assessed with a linear mixed regression model. Differences in the SNh area in fALS and sALS were assessed by the Wilcoxon test, and the relationship between SNh and ferritin, verbal fluency index and FrSBe Z-scores was assessed by Pearson’s correlation. To assess the effect of side of disease onset on the size of contralateral SNh, linear model regression was performed, which included an interaction between these covariates. An association of the demographical and clinical variables of the ALS patients with SNh area on both sides was assessed by a mixed linear regression model. In this model was assumed that SNh area depended on fixed effects (covariates) and random effects due to inter-individual variability. Firstly, a group of covariates (age, gender, family history, fALS, premorbid BMI, disease duration, phenotype, ALSFRS-R, executive or behavioural impairment, parkinsonism and SNh side) were pre-selected based on previous literature and the study goal. Finally, the combination of covariates that best fitted the model was selected according to the Akaike Information Criteria (AIC). P values of <0.05 were considered statistically significant. All the statistical analyses and graphs were performed using version 3.2.2 of the R software.

### Ethical approval

The study was approved by the Ethics Committee for Biomedical Research of the La Fe Hospital (Valencia, Spain) and has been conducted according to the principles of the Declaration of Helsinki. All the participants gave written informed consent.

### Data availability

The datasets generated during and/or analysed during the current study are available from the corresponding author on reasonable request.

## Results

### Population characteristics

One hundred and eight ALS patients (74.1% cALS, 15.7% PMA and 10.2% PLS) and 193 MD patients (102 PD and 91 with other MD) were recruited for this study. Ninety-one healthy controls, in whom at least one SNh was measureable, were also included. Overall ALS patients were moderately disabled and the time since symptoms onset ranged between 17 months in ALS to 49 months in PLS. The demographical and ultrasound characteristics of both patients and controls, and the clinical and genetic characteristics of ALS patients as per phenotype, are summarised in Table 1.

**Table 1:** Demographical, clinical and ultrasound characteristics of patients (as per phenotype) and controls.

	cALS (n = 80)	PMA (n = 17)	PLS (n = 11)	PD (n = 102)	OMD (n = 91)	Controls (n = 91)
Age (years)						
*Mean* (*SD*)	61.75 (12.26)	63.98 (13.2)	64.71 (11.68)	77.1 (88.15)	70.98 (9.58)	66.92 (12.7)
*Median* (*IQR*)	61.37 (54.93, 71.13)	65 (59.76, 72.07)	68.48 (58.95, 73.07)	70 (62.25, 74)	72 (65, 79)	69 (59, 76)
Gender (male)						
n (%)	45 (56.2%)	15 (88.2%)	8 (72.7%)	60 (58.8%)	51 (56%)	67 (73.6%)
Premorbid BMI, n = 102						
*Mean* (*SD*)	27.61 (4.53)	27.78 (4.49)	27.13 (3.64)			
*Median* (*IQR*)	27.24 (24.68, 29.76)	26.53 (24.67, 30.58)	28.07 (24.75, 29.52)			
Family history						
Dementia n (%)	10 (12.5%)	3 (17.6%)	0 (0%)			
PD n (%)	3 (3.8%)	0 (0%)	0 (0%)			
Dementia and PD n (%)	3 (3.8%)	0 (0%)	0 (0%)			
ALS or FTD n (%)	10 (12.5%)	3 (17.6%)	1 (9.09%)			
Region of onset						
Bulbar n (%)	22 (27.5%)	1 (5.9%)	3 (27.3%)			
Spinal n (%)	58 (72.5%)	16 (94.1%)	8 (72.7%)			
Side of onset or side predominance, n = 106						
Right n (%)	33 (41.2%)	8 (47.1%)	3 (27.3%)			
Left n (%)	36 (45%)	5 (29.4%)	5 (45.5%)			
Symmetric n (%)	10 (12.5%)	3 (17.6%)	3 (27.3%)			
Parkinsonism						
n (%)	4 (5%)	0 (0%)	1 (9.1%)			
Time from onset (months)						
*Mean* (*SD*)	23.46 (20.05)	42.69 (42.57)	75.38 (66.6)			
*Median* (*IQR*)	16.75 (8.78, 29.82)	25.03 (14.07, 55.8)	48.83 (37.45, 99.1)			
ALSFRS-R						
*Mean* (*SD*)	34.91 (7.74)	37.71 (6.72)	32.3 (7.21)			
*Median* (*IQR*)	35 (31, 41.25)	39 (36, 43)	34.5 (29.25, 36.75)			
UMN score						
*Mean* (*SD*)	5.23 (5.03)	0 (0)	12.4 (3.03)			
*Median* (*IQR*)	3 (1, 9)	0 (0, 0)	13.5 (12.25, 14)			
EB impairment (n = 100)						
No	46 (61.3%)	12 (80%)	3 (30%)			
Mild	24 (32%)	3 (20%)	7 (70%)			
FTD	5 (6.7%)	0 (0%)	0 (0%)			
Mutation carriers						
C9ORF72	8 (10%)	0 (0%)	1 (9.09%)			
SOD1	2 (2.5%)	3 (17.6%)	0 (0%)			
Familial ALS	11 (13.7%)	3 (17.6%)	1 (9.09%)			
Definite	2	1	1			
Probable	6	2				
Possible	3					
Right SNh area						
*Mean* (*SD*)	0.2 (0.13)	0.21 (0.1)	0.14 (0.08)	0.24 (0.13)	0.16 (0.11)	0.07 (0.08)
*Median* (*IQR*)	0.18 (0.11, 0.26)	0.19 (0.18, 0.26)	0.09 (0.08, 0.18)	0.24 (0.15, 0.3)	0.15 (0.08, 0.24)	0.06 (0, 0.11)
Left SNh area						
*Mean* (*SD*)	0.23 (0.12)	0.22 (0.12)	0.17 (0.12)	0.29 (0.14)	0.2 (0.14)	0.08 (0.09)
*Median* (*IQR*)	0.22 (0.14, 0.29)	0.2 (0.12, 0.3)	0.18 (0.06, 0.28)	0.27 (0.2, 0.38)	0.18 (0.09, 0.29)	0.06 (0, 0.12)
Left SNh area predominance						
n (%)	32 (53.3%)	6 (46.2%)	6 (75.5%)	44 (54.2%)	45 (56.2%)	40 (70.2%)
SN+, n (%)	39/71 (54.9%)	9/15 (60%)	4/11 (36.4%)	69/93 (74.2%)	31/73 (42.5%)	8 (8.8%)

ALS: amyotrophic lateral sclerosis; BMI: body mass index; cALS: classical ALS; EB: executive or behavioural; FTD: frontotemporal dementia; OMD: other movement disorders (atypical parkinsonism, vascular parkinsonism and essential tremor); PD: Parkinson’s disease; PLS: primary lateral sclerosis; PMA: progressive muscular atrophy; SNh: hyperechogenicity of substantia nigra; SN+: hyperechogenicity of substantia nigra area > 0.22 cm^2^; UMN: upper motor neuron.

### Family history and genetic analysis

Unspecified dementia was the most frequently reported family history in ALS patients, followed by ALS or FTD (Table 1). Thirteen (12%) ALS patients of 11 different families (three members of one family were included in the study) were classified as fALS as per history^18^. A pathologic expansion in *C9ORF72* was found in seven of the 11 (64%) index fALS cases. A *SOD1* mutation was found in three index fALS cases (p.E22G and p.N139H) and in two affected relatives of the index case carrying the p.N139H mutation. No mutations were identified in one fALS case. *C9ORF72* was found in two of the 95 sporadic cases (2%). Consequently, 15 ALS patients (13.9%) were, after genetic analysis, classified as fALS^18^.

### SNh area in patients and controls

It was possible to measure SNh area through the temporal acoustic bone window in at least one side in 97 (89.8%) ALS patients (including 12 of 15 fALS patients, 80%), 93 (91.2%) PD patients and 73 (80.2%) patients with other MD.

**Figure 1:**
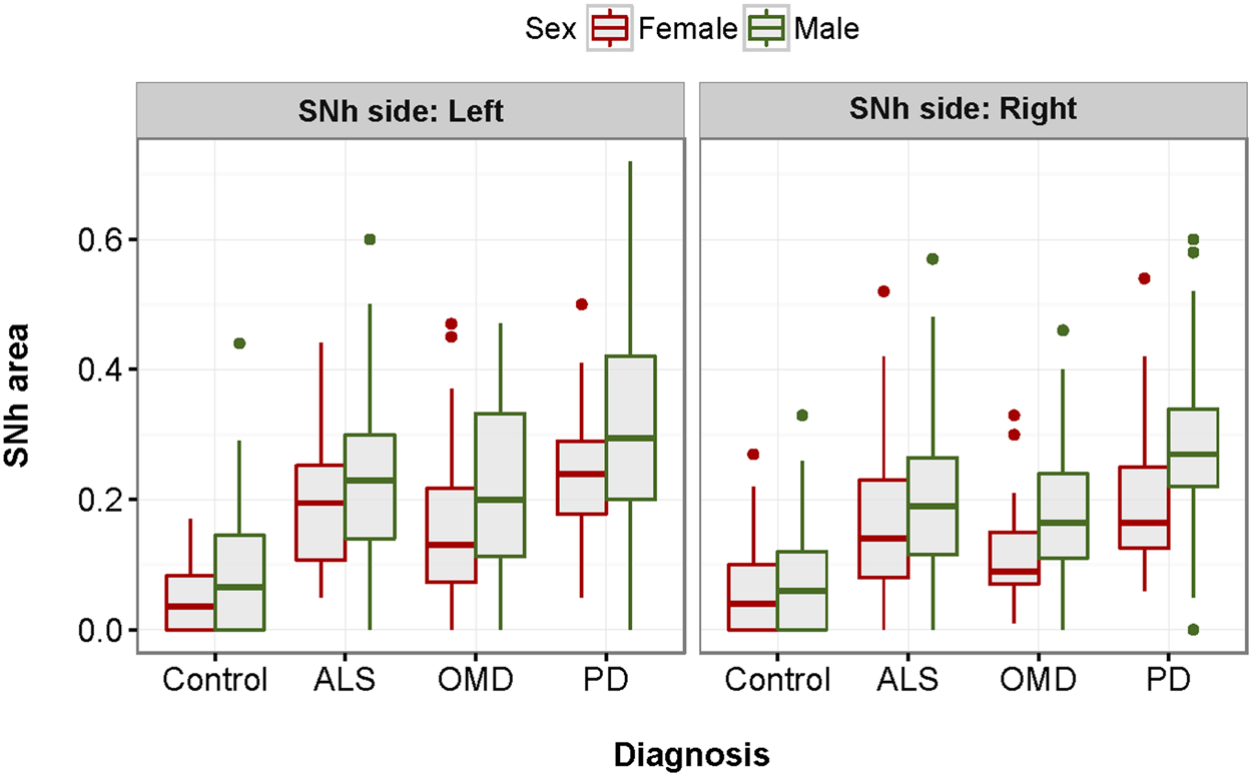
SNh area in different subgroups of patients and controls according to the SNh side and to gender. ALS: amyotrophic lateral sclerosis; OMD: other movements disorders (atypical parkinsonism, vascular parkinsonism and essential tremor); PD: Parkinson’s disease; SNh: hyperechogenicity of the substantia nigra.

cALS, PMA and PLS patients and the MD patients showed a larger SNh area than the healthy controls. The largest mean and median SNh areas were found in PD patients and the smallest ones in PLS patients (Table 1). Left SNh area was greater than the right one in all cohorts, except in PMA patients (Table 1, Fig. 1). Based on our previous report^13^, hyperechogenicity of SN (SNh+) was considered whenever one SNh area was larger than 0.22 cm^2^, whereas SNh− was considered whenever both areas were smaller than 0.22 cm^2^. Patients with only one visible SNh area, which was <0.22 cm^2^ were excluded because their classification was uncertain. Accordingly, SNh+ was found in 56.7% of ALS patients compared to 8.8% of healthy controls (Table 1).

### Contribution of demographical variables to SNh area in patients and controls

In the multivariate analysis, differences in SNh area between ALS patients and the MD controls on one side and healthy controls on the other were statistically significant after adjusting for age, gender and side of SNh (Table 2). Left SNh and male gender, but not age, were associated with a larger SNh area in the multivariate analysis (Table 2). This effect of male gender and left side was apparent in each subgroup of patients and controls (Table 1, Fig. 1).

**Table 2:** Mixed lineal regression model that analyses differences in the SNh area between controls and subgroups of patients.

	Estimate	Lower 0.95	Upper 0.95	p
ALS	0.14	0.11	0.169	< 0.001**
PD	0.196	0.167	0.225	< 0.001**
OMD	0.11	0.078	0.141	< 0.001**
Left SNh	0.029	0.016	0.042	< 0.001**
Male gender	0.047	0.025	0.07	< 0.001**
Age	0.001	0	0.002	0.135

ALS: amyotrophic lateral sclerosis; OMD: other movement disorders (atypical parkinsonism, vascular parkinsonism and essential tremor); PD: Parkinson’s disease; SNh: hyperechogenicity of substantia nigra.

### Associaton of demographical, clinical and biochemical variables with SNh area in the ALS patients

Age, gender, mutations, familial history and disease duration showed differences in the SNh+ compared to the SNh− ALS patients (Table 3). Conversely, no differences in disease characteristics were apparent, except for executive or behavioural impairment and parkinsonism, which were slightly more frequent in the SNh+ patients.

**Table 3:** Demographical, clinical and analytical characteristics of ALS patients classified according to the predefined threshold of SNh area (0.22 cm^2^).

	SN− (n = 42)	SN+(n = 55)
Age (years)		
*Mean* (*SD*)	57.35 (12.84)	62.68 (11.48)
*Median* (*IQR*)	57.45 (47.65, 68.2)	62.97 (56.54, 71.55)
Gender (male)		
n (%)	20 (62.5%)	41 (74.5%)
Body mass index, n = 102		
*Mean* (*SD*)	27.22 (4.49)	27.18 (4.3)
*Median* (*IQR*)	26.53 (24.54, 29.76)	26.53 (23.98, 29.76)
Family history	7 (16.7%)	18 (32.7%)
Dementia n (%)	1 (3.1%)	8 (14.5%)
PD n (%)	1 (3.1%)	2 (3.6%)
Dementia and PD n (%)	2 (6.2%)	0 (0%)
ALS or FTD n (%)	3 (9.4%)	8 (14.5%)
Region of onset		
Bulbar n (%)	6 (18.8%)	15 (27.3%)
Spinal n (%)	26 (81.2%)	40 (72.7%)
Parkinsonism		
n (%)	1 (2.4%)	3 (5.5%)
Time from onset (months)		
*Mean* (*SD*)	36.45 (46.83)	32.58 (32.2)
*Median* (*IQR*)	15.58 (8.43, 55.51)	21.6 (13.62, 42.77)
ALSFRS-R		
*Mean* (*SD*)	34.06 (8.55)	35.41 (7.53)
*Median* (*IQR*)	34.5 (31, 40.5)	36.5 (31.25, 41.75)
UMN score		
*Mean* (*SD*)	5.78 (5.94)	4.75 (5.11)
*Median* (*IQR*)	3.5 (0, 12.25)	2.5 (1, 9)
EB impairment (n = 80)		
No	21 (72.4%)	30 (58.8%)
Mild	8 (27.6%)	19 (37.3%)
FTD	0 (0%)	2 (3.9%)
Familial or genetic ALS, n (%)	3 (9.37%)	9 (16.36%)
C9ORF72, n (%)	1 (3.12%)	6 (10.91%)
SOD1, n (%)	1 (3.12%)	3 (5.45%)
Unknown, n (%)	1 (3.12%)	0 (0%)
Ferritin ug/L		
*Mean* (*SD*)	195.31 (155.14)	192.31 (128.31)
Median (IQR)	154 (68, 311)	155 (105.25, 240.25)

ALS: amyotrophic lateral sclerosis; BMI: body mass index; EB: executive or behavioural; FTD: frontotemporal dementia; PD: Parkinson’s disease; SNh: hyperechogenicity of the substantia nigra; SN+: area of hyperechogenicity of one substantia nigra > 0.22 cm^2^; SN−: area of hyperechogenicity of both substantia nigra ≤0.22 cm^2^; UMN: upper motor neuron.

**Figure 2:**
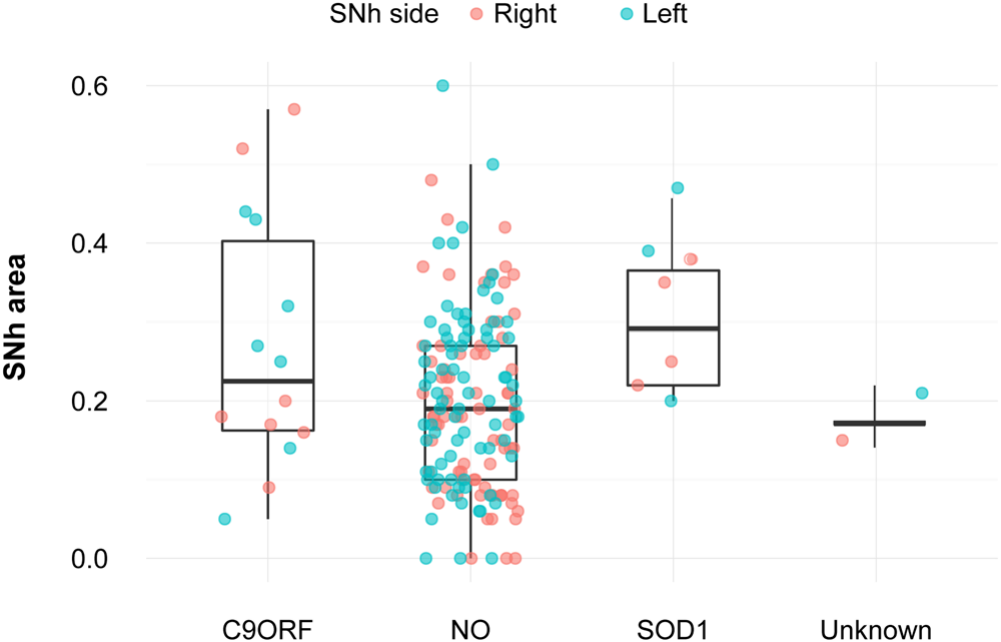
SNh area on the left and the right side in sporadic or familial ALS patients harbouring mutations (“C9ORF72” and “SOD1”), familial ALS without known mutations (“Unknown”) and sporadic patients not carrying mutations (“NO”). SNh: hyperechogenicity of the substantia nigra.

In the univariate analysis, fALS patients presented a larger SNh area than the sporadic patients (0.20 *vs*. 0.28, p = 0.01). This effect appeared to be independent of the causing mutation (Fig. 2). However, no correlation was found between the SNh and basal levels of ferritin in the ALS patients (R = −0.0005, p = 0.99) nor between SNh and verbal fluency index or any of the FRSBe subscales (Supplementary Table 1).

We found no effect of disease lateralisation on the size of contralateral SNh in ALS patients (estimate = −0.004, p = 0.91), and the left SNh area appeared to be larger independently of side of disease onset (Supplementary Table 2 and Supplementary Figure 1).

In the multivariate analysis, we first analysed all the pre-selected covariates. Subsequently, we repeated the analysis after removing those covariates that were not found to be significant in the first model, and for which there were no previous evidences of association with SNh (parkinsonism, ALSFRS-R, cognitive or behavioural impairment, disease duration and premorbid BMI). The model showed notable improvement (AIC −161 *vs*. −233). In this final model, fALS was the only variable that was significantly associated with a larger SNh area in ALS patients, but a trend to negative association for PLS and to positive association for left SNh side (Table 4) were observed.

**Tablee 4:** Mixed lineal regression model that analyses the factors that contribute to the SNh area.

	Estimate	Std. Error	Lower 95%	Upper 95%	P-value
PMA	−0.006	0.029	−0.063	0.051	0.831
PLS	−0.067	0.034	−0.135	0.001	0.055
History of PD or dementia	0.006	0.029	−0.05	0.063	0.824
Age	0.001	0.001	0	0.003	0.163
Male gender	0.036	0.023	−0.009	0.082	0.119
fALS	0.083	0.031	0.021	0.145	0.01*
Left SNh	0.022	0.012	−0.001	0.045	0.066

fALS: familial ALS; PLS: primary lateral sclerosis; PMA: progressive muscular atrophy; PD: Parkinson’s disease; SNh: hyperechogenicity of substantia nigra.

## Discussion

Firstly thought to be a marker of neuronal degeneration in the SN, more recent studies suggest the SNh role as a marker of SN vulnerability^5, 24^. Our study strongly reinforces this hypothesis and adds further evidences that genetic and constitutional factors are key to predispose to SNh in different neurodegenerative diseases and controls.

We studied a cross-sectional cohort of patients in an ALS Unit of a tertiary centre in Spain. This explains why some phenotypes (PMA and PLS) and fALS cases can be slightly overrepresented compared to prospective population-based studies. Rates of fALS and causing mutations in our cohort were similar to those previously described in European population^25^.

In our study, SNh area was found larger in all the studied diseases compared to healthy controls after adjusting for age and gender. PD patients presented the largest area, whereas patients with other MD and PLS patients had the smallest SNh area. Although an increased SNh area was first described in PD patients, it has also been found in other MD^14–16^, as well as in neurodegenerative diseases not typically characterised by SN degeneration or vulnerability, such as ALS or Friedreich’s ataxia^7, 8, 26^. Our data are consistent with those reports showing that, although PD patients are those with larger SNh, SN+ is also a frequent finding in different ALS phenotypes and other MD.

Male gender strongly associated with a larger SNh area in the entire population, and this association was apparent for each studied cohort. However, they lost significance when analysed only in ALS patients and together with other clinical variables, which also influenced SNh area. This is probably the result of having a lower statistical power due to the reduced sample size, since a trend of this effect was still evident. The effect of male gender on SNh area has been previously reported in PD and controls^5, 24, 27^, which further supports our results. Interestingly, male gender is more overrepresented in PD, cALS and PMA, but not in PLS^24, 28^. This suggests that SNh could reflect a gender-mediated differential vulnerability to certain diseases. However, this result must be taken cautiously because of the small number of studied PLS patients. Moreover, differences by gender could also be the result of a lower ultrasound penetration rate in women than men.

We found that left SNh side was larger in each cohort of patients and controls, but found no relationship between side of onset or side of symptoms predominance and the area of contralateral SNh. A larger SNh area contralateral to disease predominance has been found in PD^5^, but not in ALS^8^. Nor has SNh area been observed to correlate with the degree of clinical or subclinical impairment in PD patients, which suggests that SNh size does not actually reflect the degree of neuronal degeneration^5, 29^. Up to now, to the best of our knowledge, no study has compared the right with the left SNh area. However, in those studies reporting SNh sizes on both sides in large cohorts of PD patients and healthy controls, the reported left SNh area has almost always been larger than the right one^24, 27, 30–32^. It could be argued that side-related variations in the SNh area are explained by changes in the probe placement on the right and left side. Indeed, shifts in the position and angle of the probe can cause variations in the size of midbrain or midbrain structures. However, different studies, with diverse sonographers have found similar results^24, 27, 30–32^. Moreover, side differences remained stable when accounting for the area of the ipsilateral mesencephalon^31, 32^, suggesting that they cannot be explained by variations of the imaged midbrain. Finally, previous MRI studies in healthy individuals have also found larger iron deposits in different structures of the left brain hemisphere, including the SN^33, 34^. All this suggests that technical issues are not responsible for the differences between left and right sides. The fact that, in our study, this SNh side predominance was found in healthy individuals and patients with different diseases suggests that genetic or constitutional factors (e.g., handedness), but not disease-related factors, could underlie these differences. Interestingly, an increased dopamine metabolism has been found in the left SN^34^ and handedness seem to influence the side of onset and predominance of impairment in ALS and PD^35, 36^. Considering this and the overall predominance of right-handedness in the population, the left SNh area predominance found in our study could reflect an increased vulnerability of the left-brain hemisphere in right-handed individuals due to an increased dopamine metabolism as previously suggested^33, 34^, what ultimately results in an asymmetric onset or impairment of the disease. However, further studies in right- and left-handed patients and controls are warranted to test the influence of handedness in SNh.

In the multivariate analysis, we found that fALS, but not history of other neurodegenerative diseases, was associated with a larger SNh area in ALS patients. This effect appeared independent of the identified mutation. In PD, SNh+ has been associated with family history of PD in healthy subjects^24^ and has been found in the pre-symptomatic carriers of mutations that cause PD^5^. Furthermore, SNh+ healthy subjects have an increased risk of PD after a 5-year follow-up^37^. Altogether this suggests that SNh is a marker of genetic factors that predispose to PD^5, 24^. Our results are consistent with those in PD and suggest that the Mendelian forms of ALS are also predisposed to SNh. Conversely, family history of PD or dementia was not associated with larger SNh in ALS, suggesting that in sALS other non-genetic factors influence the SNh size.

We found no influence of ageing in SNh area. Previous studies in healthy subjects and patients have reported conflicting results^5, 24, 31, 32^. Although SNh can be found even in early childhood^32^, it seems that a mild increase in SNh area in both adolescence and very old subjects (>75 years) can occur^5, 31, 32^. However, during adulthood (30–75 years old) the SNh area remains more or less stable^5, 24, 31, 32^, which agrees with our results.

Altered ferritin levels have been found in ALS patients and associated with poor prognosis^38^ and could indirectly reflect brain iron deposits. However, we found no correlation between ferritin levels and SNh area. We neither found correlation of executive or behavioural scores with SNh area.

Low premorbid BMI has been associated with a higher risk of ALS, and genetic factors could underlie this relationship^39^. Nevertheless, we found no association between SNh and premorbid BMI either.

Finally, no association between the clinical variables (ALSFRS-R, disease duration, cognitive or behavioural impairment or parkinsonism) and SNh area was found, although very few patients exhibited parkinsonism and this result must be taken cautiously. Our results agree with previous reports that have found no association between SNh or SNh+ and ALSFRS-R, disease duration or ALS subtype (bulbar or spinal)^6–9^. Similarly, in PD, most studies have observed no association between SNh and clinical variables^5^.

### Strengths and limitations

Our study represents the largest and most thorough study of SNh in different neurodegenerative diseases and in controls conducted to date. We studied phenotypes (PMA and PLS) and variables that have not been analysed before. We also introduced several methodological improvements compared to previous studies. Firstly, most reports consider SNh to be a dichotomous variable (SNh+ and SNh−). Despite SNh+ being arbitrarily defined (percentile 90 of healthy subjects), it can be useful as a diagnostic biomarker. However, when the aim is to study the factors that contribute to SNh, dichotomising a biological variable that is in fact continuous is not methodologically sound. Secondly, in order to increase the statistical power and to avoid unnecessary data loss, we included data of both SNh sides in the inferential analysis. To date, all studies have either considered only the biggest SNh or analysed the mean SNh, which does not reflect the anatomic basis. Our data show that SNh is unequally distributed between sides. Consequently, not considering this item in inferential studies can be a source of bias. Thirdly, previous reports had studied the correlation of clinical variables and SNh in ALS, but had not accounted for confounding factors. We performed a multivariate analysis and, consequently, our results are more reliable.

However, our study also has some limitations. Firstly, the sample size for some studied variables (fALS, PLS phenotype or parkinsonism) was small as their prevalence is low, so the results on these items must be taken cautiously. Secondly, no sample size calculation was made and the overall number of included ALS patients could be insufficient for the number of studied variables. This could have limited the statistical power in the multivariate analysis and, consequently, some of the variables that show an association trend could also have some influence on SNh. Thirdly, the medical records of the ALS patients’ relatives were not systematically reviewed. This could introduce a recall bias since patients could be more likely to become aware of a family history of ALS than one of other neurodegenerative diseases. Therefore, the influence of family history of dementia and PD could have been underestimated herein. However, these limitations do not invalidate the positive results.

## Conclusions

We show that left side and male gender are risk factors for SNh in both controls and different diseases. We report for the first time that fALS and ALS-causing mutations (*C9ORF72* and *SOD1*), but not other disease-related variables, are major contributors to SNh. Finally, we demonstrate that SNh can also be found in PMA, and less frequently in PLS. Although the number of fALS, PMA and PLS patients included was small, based on our results and previous reports in both patients and healthy subjects, we hypothesise that SNh is a marker of some constitutional (male gender, handedness) or genetic vulnerability factors for neuronal degeneration that extend beyond the nigrostriatal system. Remarkably, reduced intracortical inhibition within the motor cortex, as assessed by transcranial magnetic stimulation, which is an early feature in ALS and PD^40^, has also been reported in healthy subjects with SNh^41^, further supporting this vulnerability hypothesis. Larger studies in both healthy populations and presymptomatic individuals and patients who carry ALS mutations are warranted to confirm our results and hypotheses.

## Electronic supplementary material

Supplementary Material
