# Supplementary material for: Genetic and constitutional factors are major contributors to substantia nigra hyperechogenicity
Source: Sci Rep. 2017 Aug 2;7:7119. doi: 10.1038/s41598-017-07835-z (PMC5541052; doi:10.1038/s41598-017-07835-z)
Supplement: Supplementary file 1 — Supplementary Material [file 41598_2017_7835_MOESM1_ESM.pdf]

Genetic and constitutional factors are major contributors to *substantia nigra* hyperechogenicity.

Juan F Vázquez-Costa*[1,2,3], José I Tembl[4], Victoria Fornés-Ferrer[5], Fernando Cardona[6,7], Lluis Morales-Caba[4], Gerardo Fortea[4], Jordi Pérez-Tur[6,7,8], Teresa Sevilla[1,2,3,9].

	**Pearson's r**	**CI 95%**	**P-value**
Verbal fluency index – SNh	-0.03	[-0.18, 0.13]	P = 0.73
FrSBe Apathy – SNh	-0.01	[-0.21, 0.19]	P = 0.90
FrSBe Disinhibition - SNh	0.10	[-0.10, 0.29]	P = 0.33
FrSBe Executive dysfunction – SNh	-0.05	[-0.25, 0.15]	P = 0.62
FrSBe Total - SNh	0.004	[-0.19, 0.20]	P = 0.97

Supplementary Table 1 . Correlation between several cognitive and behavioural measurements and SNh area. FrSBe: frontal systems behaviour scale; SNh: hyperechogenicity of the substantia nigra.

	**Estimate**	**Lower 0.95**	**Upper 0.95**	**p**
Left SNh	0.024	-0.032	0.08	0.4
Left side of onset	-0.028	-0.085	0.027	0.318
Left SNh: Left side of onset	-0.004	-0.083	0.075	0.913

Supplementary Table 2. Linear model regression to assess the effect of the side of disease onset on the size of contralateral SNh. Since there is no interaction between the SNh side and clinical side of onset, we concluded that side of onset does not influence SNh. SNh: hyperechogenicity of substantia nigra.

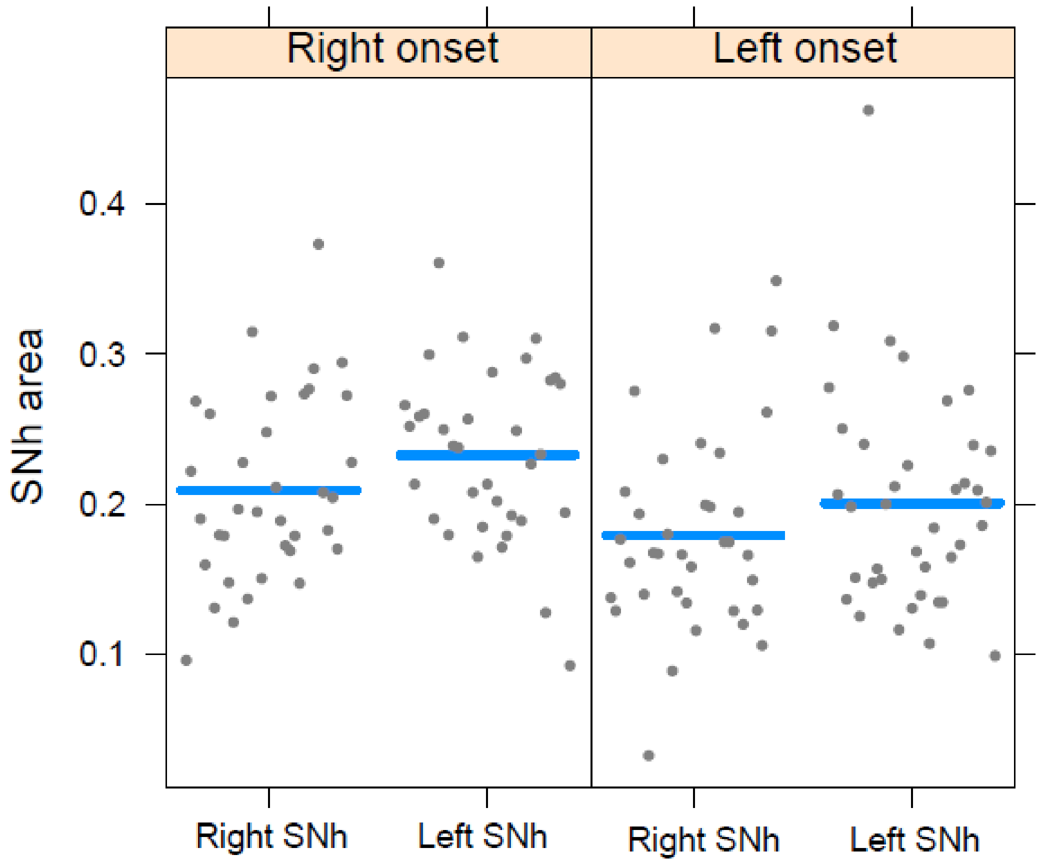

Supplementary Figure 1. Graphical representation of the right and left SNh area according to the side of disease onset. Left SNh area appears larger independently of the side of onset. SNh: hyperechogenicity of the substantia nigra.
